# Digitales Leben in der vernetzten Welt: Chancen und Risiken für die Psychiatrie

**DOI:** 10.1007/s00115-021-01203-z

**Published:** 2021-10-14

**Authors:** Andreas Meyer-Lindenberg

**Affiliations:** Zentralinstitut für Seelische Gesundheit, Klinik für Psychiatrie und Psychotherapie, J5, Medizinische Fakultät Mannheim, Universität Heidelberg, 68159 Mannheim, Deutschland

**Keywords:** Lebenswelt, Künstliche Intelligenz, Internet of things, Smartphones, Ecological momentary interventions, Environment, Artificial intelligence, Internet of things, Smartphones, Ecological momentary interventions

## Abstract

In dieser Übersicht werden Chancen und Risiken der digitalen Transformation in ihrer Bedeutung für die Diagnostik und Therapie psychischer Erkrankungen unter dem Aspekt der Konvergenz neuer digitaler Technologien thematisiert. Die Möglichkeiten Smartphone-basierter Technologien für die Erfassung des lebensweltlichen Kontextes werden erläutert und anhand zweier aktueller Forschungsergebnisse die Anwendung dieses Ansatzes auf die Untersuchung von Resilienzmechanismen zur Verbesserung der psychischen Befindlichkeit dargestellt. Im Anschluss wird die zunehmende Vernetzung des Umweltkontextes selber vor dem Hintergrund des sog. „internet of things“ (IoT) in den Blick genommen. Diese konvergierenden Technologien ermöglichen in Kombination mit neuen Entwicklungen in der künstlichen Intelligenz eine neue Generation von Interventionen in der Lebenswelt („ecological momentary interventions“, EMI), die sich auf innovative Sensoren, lokale Berechnungen des lebensweltlichen Kontextes und deren Bewertung mithilfe der künstlichen Intelligenz stützen.

## Abstract (en)

This overview addresses some opportunities and risks of digital transformation in their significance for the diagnostics and therapy of mental illnesses under the aspect of the convergence of new digital technologies. Possibilities of smartphone-based technologies for the recording of the lifeworld context are explained and the application of this approach to the investigation of resilience mechanisms for improving mental well-being is presented on the basis of two current research results. Subsequently, the increasing networking of the environmental context itself is taken into account against the background of the so-called internet of things (IoT). These converging technologies, combined with new developments in artificial intelligence, enable a new generation of ecological momentary interventions (EMI) based on innovative sensors, local assessment of the lifeworld context and their evaluation using artificial intelligence.

## Lektionen aus der Pandemie

Die COVID-19-Pandemie hat, so können wir jetzt schon sagen, wesentliche Transformationsprozesse der Lebens- und Arbeitswelt in einem nicht erwartbaren Ausmaß beschleunigt. Das bezieht sich auch auf die Arbeitswelt von Psychiatern, Psychotherapeuten und ihrer Patienten und Klienten. Die rasche und flexible Anwendung von telemedizinischen und E‑Health-Ansätzen wie Videosprechstunden hat insbesondere in den ersten Phasen der Pandemie zur Aufrechterhaltung der Versorgung gerade schwer psychisch Kranker beigetragen und ein niederschwelliges Angebot für mit den Belastungen der Lockdownsituation und sozialer Isolierung überforderten Menschen etabliert. Arbeiten von zu Hause, Konferenzen via Zoom und analoge Plattformen haben die Grenzen zwischen Arbeitswelt und Privatsphäre vermutlich dauerhaft verschoben. Vor dem Hintergrund dieser Veränderungen hat der Aspekt der digitalen Teilhabe neue Relevanz bekommen: Wer keine stabile Internetverbindung und kein Smartphone hatte oder hat, der war und ist von wesentlichen telemedizinischen Angeboten ausgeschlossen. Umgekehrt konnten viele Menschen erleben, wie wichtig Technologie zur Aufrechterhaltung sozialer Interaktionen nicht nur im medizinischen und beruflichen, sondern gerade auch im privaten Kontext sein kann. Zu Hause bleiben zu müssen, nicht reisen zu können und soziale Interaktionen nicht in der gewohnten Weise leben zu können, hat für viele von uns in neuer Weise die Wichtigkeit der (sozialen) Umwelt und ihrer engen Verbindung zum Wohlbefinden und zur psychischen Gesundheit gezeigt. Auch als Therapeuten waren die Einblicke, die uns unsere Klienten in Videosprechstunden manchmal in ihre Lebenswelt gewährt haben, oft überraschend und hilfreich.

Vor diesem Hintergrund sollen Aspekte der digitalen Transformation in ihrer Bedeutung für die Diagnostik und Therapie psychischer Erkrankungen unter dem Aspekt der Konvergenz neuer digitaler Technologien beleuchtet werden. Dabei gehen wir zunächst auf die Möglichkeiten Smartphone-basierter Technologien für die Erfassung des lebensweltlichen Kontextes ein. Die Relevanz für die Ätiopathogenese psychischer Störungen und die Erfassung des Wohlbefindens in der Lebenswelt wird an zwei aktuellen Forschungsbeispielen aus der eigenen Gruppe erläutert. Danach gehen wir auf die zunehmende Vernetzung des Umweltkontextes selber vor dem Hintergrund des sog. „internet of things“ (IoT) ein. Die Konfluenz dieser Technologien ermöglicht in Kombination mit neuen Entwicklungen in der künstlichen Intelligenz dann eine neue Generation von Interventionen, die sich auf innovative Sensoren, lokale Berechnungen des lebensweltlichen Kontextes und deren Bewertung mithilfe der künstlichen Intelligenz stützen. Abschließend sollen auf Chancen und Risiken dieser neuen technologischen Entwicklungen für Menschen mit psychischen Störungen eingegangen werden.

## Smartphone und Kontext

Eine der wesentlichen digitalen Zugänge zur Umwelt wird durch Smartphones ermöglicht. Die Anzahl der Smartphone-Besitzer ist weltweit schon sehr hoch und nimmt ständig weiter zu. Es gibt deutlich mehr Menschen, die ein Smartphone ihr eigen nennen, als eine Zahnbürste [21]. Gerade bei manchen besonders belasteten Gruppen, wie Flüchtlingen, ist das Smartphone eine der zentralen Aspekte ihrer Lebensgestaltung, Kontaktaufrechterhaltung und Teilhabe. In einem größeren Rahmen wurde dies auch in der Pandemie deutlich. Auch die Nutzung digitaler, insbesondere textbezogener/Messaging-Kommunikation bei jungen Menschen („digital natives“) ist ein wesentlicher Aspekt der sozialen Lebenswelt.

Smartphones sind einerseits wichtige Plattformtechnologien für E‑Health-Anwendungen (hierüber ist in diesem Leitthemenheft von *Der Nervenarzt* mehr in dem entsprechenden Aufsatz zu lesen). Über technologische Konvergenzen, die E‑Health-Anwendungen in der Umwelt ermöglichen oder verbessern, wird im Folgenden noch gesprochen. Hier soll zunächst ein anderer Aspekt im Fokus stehen, nämlich das Smartphone als Sensorplattform. Moderne Smartphones enthalten Dutzende von Sensoren, die beispielsweise Bewegung, Lage, Magnetfelder, Lichteinfall und Umweltgeräusche messen können. Daneben sind Smartphones nahezu grundsätzlich mit GPS(„global positioning system“)-Sensoren ausgestattet, die eine Lokalisation im Raum ermöglichen, und scannen ihr Umfeld regelmäßig für die Anwesenheit weiterer Kommunikationskanäle, wie WLAN-Masten und Bluetooth-Geräte. Diese Aspekte können in der Psychiatrie zur genauen Erfassung der lebensweltlichen Umwelt benutzt werden. Über die zugrunde liegenden Methoden haben wir unlängst berichtet [2]. Beispielhaft sollen nun zwei aktuelle Forschungsergebnisse über Resilienzfaktoren in der Umwelt dargestellt werden.

## Zwei aktuelle Forschungsbeispiele

Die Erfassung individueller Risiko- und Resilienzprofile ist eines der Kernstrategien zur besseren Personalisierung von Therapie und Prävention bei psychischen Erkrankungen. Diese aktive Forschungsrichtung, die lange vornehmlich im engeren Sinne biologische Faktoren wie beispielsweise Genetik in den Blick genommen hat, hat in den letzten Jahren zunehmend ihren Fokus auch auf die Umwelt gelegt [19]. In einer Kombination von Smartphone-basierter Erfassung der Befindlichkeit (dem sog. „ecological momentary assessment“ [EMA]) mit den durch die Sensoren ermöglichten Quantifizierungen des Kontextes liegt ein Interessenschwerpunkt unserer Arbeitsgruppe, hierbei besonders in der Erfassung von Resilienzfaktoren in der Stadtumwelt [8] – dies vor dem Hintergrund der Tatsache, dass die urbane Lebenswelt als solche nach den vorliegenden epidemiologischen Daten eher als eine Belastung für die psychische Gesundheit aufgefasst werden muss, weil häufige, aber schwerwiegende Erkrankungen, wie Depressionen und Angststörungen, bei in der Stadt Lebenden deutlich erhöht gefunden werden (in der Größenordnung um 30–50 %; [15]), schwere Erkrankungen wie die Schizophrenie bei in der Stadt Geborenen und Aufgewachsenen sogar mehr als verdoppelt sind [11]. Da die Mehrzahl der Menschen auf der Welt schon in der Stadt wohnen und sich diese Anzahl bis zum Jahre 2050 vermutlich auf mehr als zwei Drittel der Menschheit erhöhen wird, ist daher die Erfassung von Resilienzfaktoren in der Stadt dringlich. Dieses Forschungsziel verfolgen wir in der epidemiologischen longitudinalen PEZ-Studie, die in der das Zentralinstitut für Seelische Gesundheit in Mannheim umgebenden Region in einem Streifen von der Pfalz über Ludwigshafen, Mannheim und Heidelberg bis in den Odenwald hinein eine Vielfalt urbaner und ländlicher Räume in den Blick nimmt [20].

Das erste aktuelle Forschungsbeispiel aus dieser Forschungslinie bezieht sich auf die körperliche Aktivität. Hier verursachen Spezifika der Stadtlebenswelt, die „Begehbarkeit“ der Stadtteilumgebung, die Verfügbarkeit, oder eben nicht, von Nahverkehrsmöglichkeiten, Radwegen etc. große Unterschiede. In der psychiatrischen Forschung ist schon länger bekannt, dass leistungsorientiertes Training, wie man es beispielsweise in einem Fitnessstudio vornimmt, positive Effekte auf die psychische Gesundheit und auf hirnbezogene Parameter, wie die Größe des Hippokampus, hat [10]. Weniger in den Blick genommen, wenn auch vermutlich im Alltagsleben der meisten mindestens ebenso wichtig oder wichtiger, ist jedoch die nichtsportliche Aktivität („non-exercise activity“), also diejenige körperliche Betätigung, die man beim Verfolgen der üblichen Tagesbetätigungen unternimmt. Gerade diese lässt sich mithilfe des Smartphones besonders gut messen, da in diesen Geräten sowohl eine Lokalisation, via GPS, als auch eine Bewegungsmessung über in das Gerät integrierte Bewegungssensoren möglich ist. Diese Option nutzten wir in einer kürzlich erschienen Studie [17]. Wie erwartet, konnten wir dort zeigen, dass die körperliche Aktivität einen spezifischen, positiven Effekt auf das Wohlbefinden unserer Versuchsteilnehmer hatte. Während solche Phänomene der Introspektion nicht wirklich zugänglich sind, ermöglichte die Erfassung von Aktivität zusammen mit Fragen über das Wohlbefinden über den Tag verteilt eine Messung des intraindividuellen Zusammenhangs dieser beiden Phänomene. Dabei zeigte sich, dass konsistent mehr Aktivität zu einem erhöhten Gefühl, Energie zu besitzen, führte. Da diese spezifische Form des Wohlbefindens dann wiederum auch zu mehr Bewegung führt, war hier ein positiver Rückkopplungskreislauf zu erfassen, der auch in Voruntersuchungen schon gesehen wurde.

Körperliche Aktivität führt bei kleinem subgenualem Zingulum zu erhöhtem Anstieg des Energiegefühls

**Figure 1:**
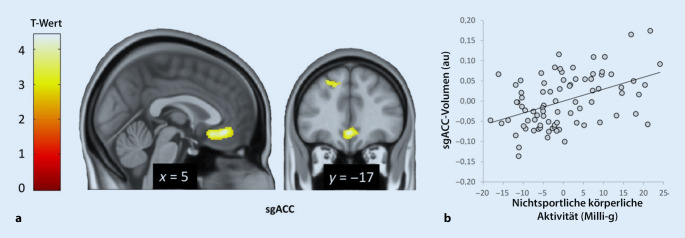

Die bei den Probanden durchgeführte Hirnbildgebung ermöglichte uns dann, diesen zwischen den einzelnen Personen in der Ausprägung unterschiedlichen Zusammenhang auf das Gehirn zu beziehen. Dabei zeigte sich sehr klar, dass dieser Zusammenhang in der Hirnstruktur abgebildet war auf einen Teil des anterioren Zingulums, genauer gesagt, das subgenuale Zingulum (Abb. 1). Das ist nun von besonderem Interesse, da dieser Teil des Zingulums in einer Reihe vorheriger Studien mit affektiven Störungen, insbesondere Depressionen, und ihrer Behandlung in Beziehung gebracht worden ist. Ein kleineres subgenuales Zingulum ist dabei ein Faktor, der ein erhöhtes Erkrankungsrisiko und ein schlechteres Ansprechen auf antidepressive Therapie, Psychotherapie, tiefe Hirnstimulation und Elektrokrampftherapie vorrausagt, also auch auf das ganze Spektrum der üblicherweise angewandten Therapien. Wir fanden nun interessanterweise, dass Versuchsteilnehmer mit einem kleineren subgenualen Zingulum einerseits eine Tendenz hatten, bei weniger Bewegung im Alltag mit einer größeren Reduktion ihrer Energie zu reagieren. Dies ist im Zusammenhang der Depression plausibel, da insbesondere zu Beginn einer Episode eine Verminderung des Antriebs auf diese Weise zu einer Minderung der Energie und dadurch im Kontext des oben geschilderten Rückkopplungskreislaufs zu einem noch stärkeren Abfall des Antriebs führen würde. Interessanterweise war aber auch zu sehen, dass über dem Durchschnitt liegende körperliche Aktivität bei diesen Probanden sogar zu einem höheren Anstieg des Energiegefühls führte als das bei der Vergleichsgruppe mit einem größeren subgenualen Zingulum der Fall war. Dies legt nahe, dass gerade bei dieser nach den erwähnten Voruntersuchungen schwierig zu behandelnden Gruppe eine explizite Integration bewegungsförderlicher Maßnahmen im Alltag in das Therapieportfolio hilfreich sein könnte.

**Figure 2:**
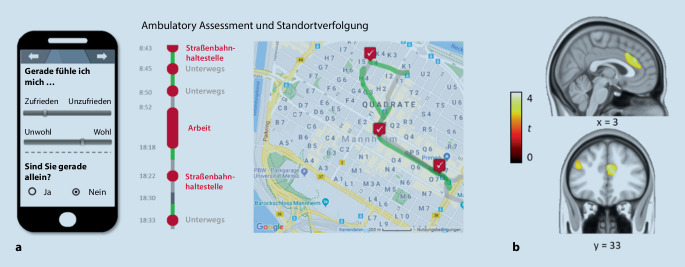

Als zweites Beispiel soll die Erfassung sozialer Interaktionen dienen. Hier ist gerade durch die Pandemie vielen noch einmal klargeworden, wie wichtig soziale Interaktionen im Alltag für unser Wohlbefinden sind. Dem korrespondiert eine große Anzahl von Studien, die das supportive soziale Netzwerk als eine der zentralen Determinanten somatischer und psychischer Gesundheit zeigen [7]. In der hier vorgestellten, jüngst in *JAMA Psychiatry* erschienen Studie [3] benutzten wir Smartphones, um Probanden während des Alltags zu fragen, ob und wie sie sozial interagierten, und setzten dies in Beziehung zu ihrem Wohlbefinden. Wie zu erwarten war, ergab sich eine deutliche positive Beziehung zwischen Wohlbefinden und sozialen Interaktionen. Ähnlich wie oben für die körperliche Aktivität dargestellt, variierte diese Beziehung intraindividuell jedoch stark von Versuchsteilnehmer zu Versuchsteilnehmer. Dieses Ausmaß, in dem soziale Interaktionen positiv auf das Wohlbefinden einwirkte, bezogen wir nun wiederum auf die Hirnstruktur. Hierbei fand sich ein deutlicher Zusammenhang mit einer anderen Subregion des Gyrus cinguli, am Übergangsbereich des sog. „emotionalen“ und „kognitiven“ Zingulums (Abb. 2). Diese eng miteinander verknüpften Subregionen sind in einer Vielzahl von Voruntersuchungen mit zentralen Aspekten der sozialen Interaktion und der Emotionsregulation verknüpft worden, „passend“ also zu dem hier untersuchten Phänomen, ebenso wie es oben für Depression und das subgenuale Zingulum dargestellt wurde.

Die digitale Erfassung lebensweltlicher Aspekte über die Smartphone-Plattform ermöglicht also, wie diese beiden aktuellen Forschungsergebnisse zeigen, die Untersuchung von für die Psychiatrie zentralen Aspekte intraindividueller Variabilität, die dann wiederum rückbezogen werden kann auf Mechanismen wie in unserem Beispiel der Hirnstruktur. Solche Ansätze werden in der nahen Zukunft eine sprunghafte Ausweitung ihres Erfassungs- und Anwendungsgebietes durch eine Digitalisierung der Umwelt selber erfahren; das Stichwort hier ist das des „internet of things“ (IoT).

## Der Kontext vernetzt sich: IoT

Das Internet der Dinge („internet of things“) ist ein Sammelbegriff für eine Gruppe von Technologien, die gegenwärtig eine globale Infrastruktur für die digitale Vernetzung sowohl physischer als auch rein digitaler Objekte schafft und damit ermöglicht, sie durch entsprechende Informations- und Kommunikationstechniken zusammenarbeiten zu lassen. Hierfür sind Objekte mit immer mehr Sensoren und kleinen eingebetteten Computern ausgestattet, deren Vernetzung durch eine entsprechend leistungsfähige Architektur, insbesondere „5G“, ermöglicht wird. Während traditionellerweise die Information solcher Sensoren zentral zusammengeführt und verarbeitet wird, ist im Zentrum des Interesses beim IoT auch die lokale Informationsverarbeitung, einschließlich des sog. „computing on the edge“.

Was heißt das nun für die Psychiatrie? Wie ein kürzlich erschienener Review [4] aufzeigt, ergibt sich eine ganze Reihe von Anwendungen in der Erfassung und Modifikation von Befindlichkeit und Verhalten bei schweren psychischen Erkrankungen einschließlich Depression, bipolarer Störung und Schizophrenie, für die jeweils schon mehrere Studien vorliegen. Als Anwendungsbeispiel sei hier das Thema der sozialen Interaktion fortgeführt. Während wir unter der gegenwärtigen technologischen Situation noch erfragen müssen, ob unsere Versuchsteilnehmer in der Lebenswelt soziale Interaktion hatten, ermöglicht das Fortschreiten des IoT hier technologiebasierte, passive und kontinuierliche Messmöglichkeiten. Schon heute kann über die Erfassung von Bluetooth-Geräten in der Umgebung eine grobe Einschätzung der sich um eine Person herum befindlichen Personen gemacht werden. Dieses Signal ist jedoch nur eingeschränkt verwertbar, da zahlreiche Menschen die Bluetooth-Funktion ihrer Geräte nicht angeschaltet haben, um Batterie zu sparen und die Verortung des Bluetooth-Signals im Raum nahezu nicht möglich ist. Dennoch lassen sich bereits mit dieser Methodik schon Aufschlüsse über soziale Netzwerke ziehen. Auch im Rahmen der Corona-App wird, wie bekannt, eine ähnliche Technologie angewandt, um Nähe zwischen zwei Menschen zu erfassen und ggf. infektionsepidemiologisch relevante Informationen auszutauschen. Neue Sensoren in der Umgebung ermöglichen hier jedoch einen besseren Zugang. Hierbei geht es insbesondere um sog. RFID(„radio-frequency identification“)-Sensoren, die in großer räumlicher Exaktheit die relative Position und Distanz von Menschen erfassen können. Solche Sensoren sind bereits in vielen Smartphones vorhanden und können im Rahmen des „internet of things“ mit der räumlichen Umgebung so verknüpft werden, dass sich hieraus eine passive Messung sozialer Interaktion ermöglicht. Wie wir kürzlich dargestellt haben [6], wenden wir eine solche Technologie zur Erfassung der Effekte der therapeutischen Gemeinschaft in der ZI-Soteria bereits an. Hierzu ist dieser Bereich des Zentralinstituts (ZI) entsprechend instrumentiert. Im Rahmen des IoT werden solche Ressourcen der Digitalisierung jedoch zumindest in großen urbanen Umfelden flächendeckend vorhanden sein.

Digitale Assistenten können suizidale Äußerungen erkennen und darauf reagieren

Diese technologischen Möglichkeiten und ihre Implikationen für die Psychiatrie werden im Rahmen des Ausbaus des IoT sprunghaft anwachsen. Ende 2019 gab es bereits 3,6 Mrd. über das IoT vernetzte Objekte, eine Zahl, die sprunghaft durch die flächendeckende Einführung von 5G ansteigen wird. Längst sind es nicht nur Industrieroboter und Computer, sondern Haushaltsgeräte und sogar Kleidungsstücke, die mit entsprechenden Sensoren ausgestattet sind. Die Kommunikation mit diesen Dingen verlagert sich entsprechend von Tastaturen und Knöpfen zunehmend in andere Interaktionsformen, insbesondere in sprachliche Kommunikation, die Entwicklung von „voice-user-interfaces“ (VUI) schreitet rapide voran, und schon heute sind digitale Assistenten wie Alexa oder Siri darauf ausgerichtet, suizidale Äußerungen ihrer Benutzer zu erkennen und darauf mit entsprechenden Hilfsangeboten zu reagieren [13].

Ein zentraler Nutznießer dieser technologischen Umwälzungen werden Stadtbewohner sein. Die Dichtigkeit der Stadt bedingt und ermöglicht besondere Vorzüge, die durch die Art und Vernetzung dieser Sensoren zu heben sind. Das „Smart-City“-Konzept kann, gerade wenn es sich mit der sensorbezogenen Erfassung des Wohlbefindens und der psychischen Gesundheit vernetzt, in letzter Konsequenz zu einer adaptativen Stadt führen, in der Umweltrahmenbedingungen, die einen negativen Einfluss auf die Befindlichkeit haben, man denke an lokalen Lärm oder Staub, auch in die Planung und Steuerung einbezogen werden können.

## Konfluenz von Technologien: digitale Sensoren und „artificial intelligence“

Während solche qualitativen Veränderungen allein schon durch die Anzahl und Art der Sensoren sowie ihre Vernetzung untereinander zu erwarten sind, ergibt sich ein weiterer Sprungfortschritt durch die Kombination dieser großen Datensätze mit den Möglichkeiten der künstlichen Intelligenz. Diese hatten wir vor einiger Zeit dargestellt [12]. Gerade die Verbindung von maschinellem Lernen mit lokaler Informationsverarbeitung im Sinne des „computing on the edge“ ermöglicht prinzipiell neue Wege, die lokale Befindlichkeit nicht nur zu erfassen, sondern auch therapeutisch zu reagieren. Dieses als „ecological momentary interventions“ bezeichnete Feld hatten wir in einem narrativen Review kürzlich im Kontext der stationsäquivalenten Behandlung psychisch Kranker untersucht [16]. „Ecological momentary interventions“ (EMI) können evidenzbasierte Interventionskomponenten auf die jeweiligen lokalen Bedürfnisse der Patienten in ihrem Kontext adaptieren und, beispielsweise per Text-Message, auch in der Lebenswelt implementieren [14]. Dafür können sie auf den beschriebenen Möglichkeiten der sensorbasierten Erfassung aufbauen.

Erste Ergebnisse bei jungen Menschen, beispielsweise im Kontext des EMI-Compass-Projekts, sind durchaus vielversprechend [16], auch zeigt sich, dass die Akzeptanz solcher Ansätze zumindest bei den von uns behandelten jungen Menschen hoch ist. EMI-Compass untersucht die Machbarkeit und Wirksamkeit einer ambulatorischen, mitgefühlsorientierten Intervention zur Verbesserung der emotionalen Resilienz bei hilfesuchenden Jugendlichen, denn die Verbesserung der Stressverarbeitung im Jugendalter ist eine vielversprechende Strategie zur Prävention schwerwiegender psychischer Probleme im späteren Leben. Sicherlich ist, wie auch im Beitrag über E‑Health in dieser Ausgabe von *Der Nervenarzt* dargestellt, die notwendige Evidenzbasis hierfür oft noch zu schaffen. Eine randomisierte klinische Studie am ZI widmet sich beispielsweise der Acceptance- und Commitment-Therapie (ACT), die auf die Verbesserung des flexiblen Umgangs mit psychischen Symptomen im Vorfeld einer schweren psychischen Erkrankung abzielt, in diesem Kontext [22].

Über die Analyse von Sprachstruktur können therapierelevante Parameter erfasst werden

Hier erhoffen wir uns neue Impulse aus der künstlichen Intelligenz insbesondere deshalb, weil, auch wenn die „big-data“ aus der Sensorumgebung noch so gut aufbereitet sind, am anderen Ende der therapeutischen Intervention die Selektion von Interventionen im Einzelfall in der Zukunft nicht immer durch einen menschlichen Therapeuten erfolgen muss. Hier kann die Auswahl unter einem vorab zwischen Therapeut und Patient festgelegten Spektrum von Interventionen durchaus mit Methoden der künstlichen Intelligenz erfolgen. Die hierbei zu beachtenden ethischen und auch medizinrechtlichen Probleme sind zum Teil erheblich und erfordern eine sorgfältige Planung der Therapie, auch um die Patientensicherheit zu gewährleisten [12]. Neben Methoden des maschinellen Lernens mithilfe „deep neural networks“ im Allgemeinen [1] sind dabei auch neue Entwicklungen der semantischen künstlichen Intelligenz zu erwähnen, die über eine Erfassung von Sprachstruktur diagnosebezogene und potenziell therapierelevante Parameter aus der gesprochenen und der geschriebenen Sprache, wie sie beide in der Lebenswelt ganz üblicher Weise über Smartphones weitergeleitet werden, erfassen können [18]. Hier dürfte sich unserer Einschätzung nach in der Zukunft ein wesentliches psychiatrisches Anwendungsfeld ergeben. Hier existieren intensive Forschungsanstrengungen, beispielhaft sei das Relater-Projekt genannt (https://www.gesundheitsforschung-bmbf.de/de/relater-verbesserung-der-kommunikation-bei-der-psychiatrischen-versorgung-von-gefluchteten-8807.php), das sich im Rahmen der Forschungsverbünde zur psychischen Gesundheit geflüchteter Menschen der Spracherfassung und maschinellen Übersetzung bei Geflüchteten widmet, die Kontakt zum psychiatrischen Versorgungssystem suchen.

## Chancen und Risiken für Menschen mit psychischen Störungen

Wie jede Sprunginnovation werfen diese Entwicklungen nicht nur Chancen für die Therapie und Diagnostik für die Menschen mit psychischen Störungen auf, sondern auch gravierende ethische Fragen und Risiken. Die hier in Rede stehenden persönlichen Daten sind oft hoch privat und bedürfen daher des besonderen Schutzes der betroffenen Menschen. In Studien sind Fragen der Einverständniserklärung, Anonymisierung und Pseudonymisierung und Datensicherheit von großer Wichtigkeit. Im Alltagsleben sind die Persönlichkeitsrechte beispielsweise im Rahmen der Datenschutz-Grundverordnung (DSGVO), die die General Data Protection Regulation (GDPR) der EU implementiert, deutlich besser geschützt als noch vor wenigen Jahren, hier ergeben sich jedoch in einer durchgehend digitalisieren vernetzten (GDPR) Umwelt natürlich entsprechend sehr weitreichende Möglichkeiten, solche Schutzrechte zu umgehen. Umgekehrt wird die Möglichkeit zur Teilnahme an der digitalen Lebenswelt auch ein wesentlicher Aspekt der Teilhabe sein. Auch hier ermöglicht die Technologie zahlreiche Möglichkeiten, so wie sie beispielsweise gegenwärtig im Bereich der augmentierten Realität zur Verbesserung der Teilhabe von Menschen mit psychiatrischen Beeinträchtigungen erforscht werden. Dabei kann unter anderem in spielerischer Form das Interesse an menschlichen Gesichtern in der Umgebung belohnt werden oder die Erkennung von Emotionen aus Gesichtsausdrücken durch entsprechende Markierungen erleichtert werden [9]. Natürlich birgt dieser Ansatz auch Risiken, man denke beispielsweise an die Perpetuierung und Vergröberung von Stereotypien über psychische Erkrankungen im Zusammenhang sozialer Netzwerke. Am Beispiel der Schizophrenie kann man zeigen, dass stigmatisierende Online-Posts entsprechend stigmatisierende Antworten nach sich ziehen im Sinne eines Echokammereffekts [5]. Den diskutierten Vorzügen von Online- und mobilen Therapien stehen Risiken der verminderten Qualität der Arzt-Patienten-Beziehung gegenüber. Sicherlich wird es wichtig sein, durch die niederschwellige Bereitstellung entsprechender Technologien, wie Smartphones, und einem Ausbau des Breitbandnetzes über auch entlegenere Bereiche der Bundesrepublik Deutschland die Teilhabe technologisch zu ermöglichen. Wie wichtig das ist, hat ja wiederum die COVID-19-Pandemie gezeigt.

Psychisch kranke Menschen dürfen nicht von den neuen Möglichkeiten „abgehängt“ werden

Unbedingt vermieden werden muss, dass schwer psychische erkrankte Menschen mit unzureichender „digital literacy“ von diesen neuen Möglichkeiten „abgehängt“ werden. Eine Förderung der digitalen Kompetenz schwer psychisch erkrankter Menschen sollte daher ein Aspekt der Förderung von Therapie und Teilhabe bzw. der Rehabilitation werden.

Verschwiegen sei auch nicht, dass sich auch im technologischen Kontext noch erhebliche Probleme beispielweise der Standardisierung oder der Interoperativität ergeben. Im engeren medizinischen Kontext sind großvolumige Förderinstrumente wie beispielsweise die Medizininformatikinitiative damit befasst, die Zusammenführung klinischer Daten über Standorte und die Zusammenführung klinischer und wissenschaftlicher Daten zu ermöglichen und den betroffenen Patienten die Daten auch zugänglich zu machen sowie ihre Persönlichkeitsrechte zu schützen. Auch hier ergeben sich noch weite Strecken zwischen dem Potenzial und der Ambition der Technologien und dem, was im realen Versorgungsumfeld gegenwärtig möglich ist. Sicherlich positiv zu sehen ist unter den genannten transformativen Aspekten dieser Technologien eine regulatorische Strukturierung des stetig anwachsenden Angebots an E‑Health-Anwendungen wie Apps. Hierzu wird in dem korrespondierenden Artikel in dieser Ausgabe von *Der Nervenarzt* Weiteres ausgeführt.

Auch unter Inrechnungstellung dieser Risiken sind die Chancen für die Verbesserung von Prävention, Diagnostik und Frühintervention bei gerade schweren psychischen Erkrankungen durch diese technologischen Möglichkeiten erheblich; es bleibt zu hoffen, dass das regulatorische, rechtlich-ethische und auch therapeutische Umfeld diese Möglichkeiten ergreift und sorgfältig im Interesse der Betroffenen und ihrer Angehörigen umsetzt.

## Fazit für die Praxis

- Technologische Möglichkeiten, die sich aus der Sensorbestückung und Lokalisierung moderner Smartphones ergeben, ermöglichen die Erfassung des lebensweltlichen Kontextes und dessen Einfluss auf die Befindlichkeit in neuem Ausmaß. Wie aktuelle Forschungsbeispiele zeigen, lassen sich so relevante Resilienz- und Risikofaktoren in ihrem inneren Einfluss auf die Betroffenen analysieren.
- In Verbindung mit technologischen Entwicklungen wie dem „internet of things“ und der künstlichen Intelligenz kann hierauf aufbauend eine neue Generation ökologischer Interventionen in der Lebenswelt etabliert werden, die gerade für schwere psychische Erkrankungen besonderes Potenzial bietet. Hierbei sind jedoch gravierende Aspekte des Datenschutzes, der Privatsphäre sowie des regulatorischen und medizinethischen Umfelds zu bedenken und kontinuierlich anzupassen.
